# N-acetylcysteine *versus* Dopamine to Prevent Acute Kidney Injury after Cardiac Surgery in Patients with Preexisting Moderate Renal Insufficiency

**DOI:** 10.21470/1678-9741-2016-0028

**Published:** 2017

**Authors:** Omer Faruk Savluk, Fusun Guzelmeric, Yasemin Yavuz, Deniz Cevirme, Emre Gurcu, Halide Ogus, Tulay Orki, Tuncer Kocak

**Affiliations:** 1 Kartal Kosuyolu High Training and Education Hospital, Istanbul, Turkey.

**Keywords:** Acute Kidney Injury/Prevention & Control, Cardiac Surgical Procedures, Acetylcysteine, Dopamine

## Abstract

**Objective:**

Acute kidney injury after cardiac surgery is associated with mortality and morbidity. Therefore, strategies to prevent acute kidney injury are very important. The aim of this placebo-controlled randomized double-blind study was to compare the prophylactic efficacy of N-Acetylcysteine and dopamine administration in patients with pre-existing moderate renal insufficiency who were undergoing cardiopulmonary bypass.

**Methods:**

This study included 135 patients with pre-existing moderate renal insufficiency who were scheduled for coronary artery bypass grafting surgery. Serum creatinine and GFR were recorded preoperatively and on the first and second postoperative days.

**Results:**

On the first and second postoperative days, the drugs used showed statistically significant differences among the creatinine groups (*P*<0.001). According to Tukey’s HSD, on the first and second PO, the creatinine of Group N, D and P were significantly different (*P*<0.001). On the first and second PO, the used drugs showed statistically significant differences among the effects of eGFR (*P*<0.001). According to Tukey’s HSD on the first postoperative day, the average eGFR score of Group N compared to D and P were significantly difference (*P*<0.001). On the second postoperative day, the eGFR of Group N and D showed no difference (*P*=0.37), but P showed a difference (*P*<0.001).

**Conclusion:**

We found that the prophylactic use of intravenous N-Acetylcysteine had a protective effect on renal function, whereas the application of renal dose dopamine did not have a protective effect in patients with pre-existing moderate renal failure.

**Table:** 

Abbreviations, acronyms & symbols	
AKI CABG CPB CVP ECG EF eGFR GFR MAP NAC NGAL ROS	= Acute kidney injury = Coronary artery bypass graft = Cardiopulmonary bypass = Central venous pressure = Electrocardiogram = Ejection fraction = Estimated glomerular filtration rate = Glomerular filtration rate = Mean arterial pressure = N-acetylcysteine = Neutrophil gelatinase associated lipocalin = Reactive oxygen species

## INTRODUCTION

Acute kidney injury (AKI) after cardiac surgery is associated with mortality and morbidity^[1]^. In recent years, patients undergoing cardiac surgery have been increasingly older, and they have experienced co-morbidity more frequently. Therefore, strategies to prevent AKI are extremely important. The pathophysiology of AKI in patients undergoing cardiac surgery is multifactorial and includes hemodynamic factors that could lead to kidney hypoperfusion, effects of nephrotoxic drugs and consequences of the systemic inflammatory reaction induced by cardiopulmonary bypass (CPB). These factors may contribute to renal ischemia and systemic hypoxic inflammation. During CPB, nonpulsatile flow and renal perfusion are reduced by approximately 30%, while mean arterial pressure decreases. While patients with normal renal function can tolerate this without developing any clinical disorders, it can be a problem for patients with preexisting renal dysfunction.

Several drugs have been used for renal protection during cardiac operations, including dopamine, mannitol, diuretics, fenoldopam, enalaprilat, dexamethasone, and diltiazem^[2]^. N-acetylcysteine (NAC) is an alternative therapy that may prevent perioperative kidney injury. Due to its anti-inflammatory and antioxidant properties, NAC attenuates several mechanisms of kidney injury during cardiac surgery, namely the systemic inflammatory response, free radical injury and ischemia^[3]^. The effects of dopamine include an increase in renal blood flow via activation of dopaminergic receptors in the renal vasculature^[4]^.

The aim of this placebo-controlled randomized double-blind study was to compare the prophylactic effects of NAC and dopamine administration in patients with preexisting mild renal insufficiency undergoing coronary bypass using CPB on renal function.

## METHODS

### Patients Population and Study Design

The institutional local ethics committee approved the protocol and informed consent was obtained from each patient. This study was designed as a placebo-controlled randomized double-blind study. The study included 135 patients with preexisting moderate renal insufficiency who were scheduled for elective coronary artery bypass grafting surgery (CABG) with CPB.

The inclusion criteria were as follows: 1) age > 18 years; 2) preexisting moderate renal insufficiency [glomerular filtration rate (GFR) < 60], with moderate renal insufficiency defined as an estimated glomerular filtration rate (eGFR) (Cockcroft-Gault equation) < 60 ml/min; and 3) CABG surgery with CPB.

The exclusion criteria were as follows: 1) severe preexisting renal insufficiency; 2) multiorgan failure; 3) preoperative hemodynamic instability (patients receiving intra-aortic balloon pump support and vasoactive therapy); 4) planned off-pump surgery; 5) planned deep hypothermic circulatory arrest; 6) previous cardiac surgery; 7) previous operation under emergency conditions; 8) severe heart failure [patients with ejection fraction (EF) < 35%]; 9) diabetes mellitus; and 10) allergies to NAC.

### Randomization

The patients were randomly divided into three groups, as follows: Group N, 45 patients receiving NAC; Group D, 45 patients receiving renal-dose dopamine (2.5 mcg/kg/min); and Group P, 45 patients comprising the control group. In Group N, 50 mg/kg of NAC was administered as a loading dose in 100 cc of 0.9% NaCl for 15 minutes, and then 20 mg/kg/h of NAC was provided as an infusion in 100 cc of 0.9% NaCl during the operation. In Group D, 2.5 mcg/kg/min of dopamine (renal dose) was initiated after induction and given during the operation (400 mg dopamine in 100 cc of 0.9% NaCl). Finally, in Group P, the control group, 100 cc of 0.9% NaCl was administered after induction for 15 minutes as a placebo.

Randomization assignment of patients to Groups N, D, and P was performed with a list of random numbers generated by a computer program’s random function. The list contained the natural numbers 1, 2, and 3. These numbers were allocated as follows: 1, Group N; 2, Group D; and 3, Group P. The allocation into the treatment or the placebo group and the preparation of the study drug were performed by an individual who as unrelated to the study. Personnel, patients, and the individual participating in the data collection and data analysis were blinded to the treatment assignments.

### Anesthesia Management

The patients were evaluated preoperatively 1 day before surgery. Electrocardiogram (ECG), pulse oximetry, cannulation of the invasive arterial pressure (20 gauge) from the left radial artery, and peripheral venous access (18 gauge) from the right arm were inserted into the patient, who was taken to the operating room on the day of the surgery. After induction of anesthesia and intubation, an 8F central venous catheter was inserted from the right internal jugular vein. Midazolam (0.05 mg/kg), propofol (2 mg/kg), fentanyl (5 mcg/kg), and rocuronium (0.6 mg/kg) were used for the induction of anesthesia. Anesthesia was maintained with a mixture of 2% sevoflurane, 60% oxygen, and 40% air. In addition, 0.2 mg/kg of rocuronium, 0.02 mg/kg of midazolam, and 1 mcg/kg of fentanyl were applied every 30 minutes. All patients were ventilated with positive pressure. Ventilation parameters were set as a tidal volume of 10 ml/kg and a respiratory rate of 10-12/min, which was assessed by arterial blood gases. All patients received 500 ml ringer lactate before the induction of anesthesia. Thereafter, IV ringer lactate solution was infused to keep the central venous pressure (CVP) at 10-12 mmHg.

### Cardiopulmonary Bypass Management

Before cannulation for CPB, 3 mg/kg of heparin was administered for all patients. When the activated coagulation time was > 400/sec, it was passed to the pump. Nonpulsatile CPB flow rates of 2-2.4 L/min/m^2^ were applied, and the mean arterial pressure (MAP) was kept at 50-60 mmHg. Hematocrit concentration was maintained at 25-28%. Moreover, mild hypothermia (28-30°C) was reached during CPB. Myocardial protection was achieved with intermittent antegrade. A temperature of 37°C was achieved after surgery, and the patient was removed from CPB. Following this, coagulation was provided with protamine; once hemodynamic stabilization (MAP 70-90 mmHg) was achieved, patients were brought to the intensive care unit.

### Data

Demographic (age, sex, and weight) and perioperative data (operation time, cross-clamp time, EF, urine volume, and total amount of hydration) were recorded. In addition, the side effects of NAC (rash, wheezing, hemolysis, and neutropenia) were monitored continuously.

Serum creatinine and GFR were recorded preoperatively and on the first and second postoperative days. GFR was calculated using the Cockcroft-Gault formula (eGFR=[140- age] *weight/72 *SCr [mg/dL]).

### Statistical Evaluation

All data were analyzed using SPSS 16.0 software. All groups were evaluated by one-way analysis of variance (ANOVA) with repeated measurements. A *P*-value < 0.05 was considered statistically significant. The categorical values were evaluated using a chi-square test, and *P*-values < 0.05 were considered statistically significant. Tukey’s HSD of the *post hoc* test was performed for comparison to determine the source of this difference between the groups.

## RESULTS

Between March 2013 and March 2014, 135 patients with preexisting moderate renal insufficiency were divided randomly into three groups and given NAC (n=46), renal-dose dopamine (2.5 mcg/kg/min; n=45), or a placebo (n=44). After randomization, at the discretion of the attending surgeon, five patients underwent unplanned off-pump surgery (three patients in the Group N and two in Group D). Four patients underwent unplanned circulatory arrest (two in Group N, one in Group D, and one in Group P). Six patients underwent reoperation (one in Group N, two in Group D, and three patients in Group P). Thus, the statistical analyses included 120 participants. The patients’ characteristic is presented in Table 1 and operative variables are presented in Table 2 for each group. No statistically significant differences among Groups N, D, and P were identified with respect to the demographic characteristics or operative variables (*P*>0.05). Moreover, there were no differences in the total amount of fluids given, but urine outputs exhibited statistically significant differences among Groups N, D, and P, with Group D showing a significant increase.

**Table 1:** Patient characteristics.

Variables	Group N	Group D	Group P	*P*
Age (years)	63±5.9	62±5.8	63±4.3	0.17
Weight (kg)	79.3±13	81±15	77±14	0.87
Sex (men)	24 (60%)	22 (55%)	19 (47.5%)	0.63
Ejection fraction (%)	53±12	56±9	55±10	0.83

**Table 2:** Intraoperative variables.

Variables	Group N	Group D	Group P	*P*
Operation period (min)	253±42.1	255±39.7	254±40.5	0.35
Aortic cross clamp period (min)	46±17.3	48±15.5	49±14.3	0.17
Cardiopulmonary bypass (min)	73±23.2	76±33.2	74±28	0.29
Urine output during operation (ml)	575.6±281	912.3±535	623.6±337	<0.001
Total amount of hydration (ml)	1755±128	1823±193	1712±154	0.85
Blood transfusion (n)	12 (30%)	10 (25%)	10 (25%)	0.24
Central venous pressure (cmH_2_0)	7.49±1.56	8.39±2.15	7.58±2.37	0.19
Mean arterial pressure (mmHg)*	58.15±5.24	59.83±7.73	57.35±6.61	0.21

* Lowest value on bypass

None of the patients presented side effects of NAC (rash, wheezing, superficial phlebitis, hemolysis, or neutropenia). In addition, no patients required dialysis in the postoperative period.

**Fig. 1:**
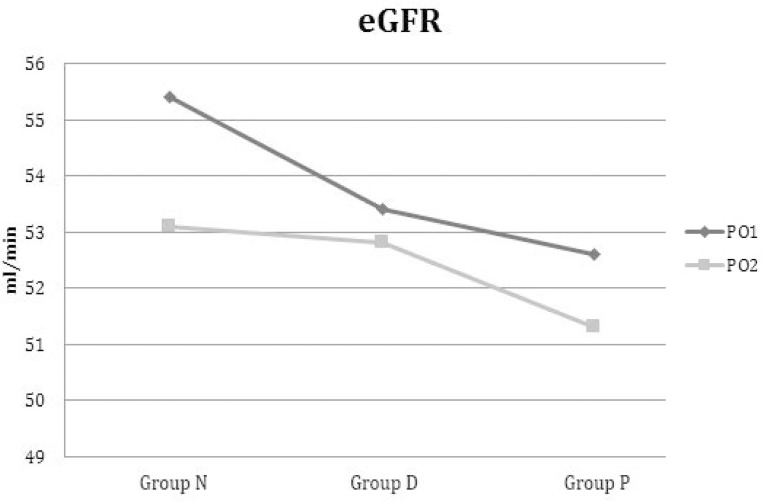
Evolution of eGFR from first postoperative day to second postoperative day in NAC, Dopamine and Placebo Groups.

Preoperative eGFR showed no statistically significant differences among Groups N, D, and P (*P*=0.43). In the first and second postoperative periods, the used drugs showed statistically significant differences in terms of the effects of eGFR (*P*<0.001; Table 3). A *post hoc* test was performed for comparison to determine the source of this difference between the groups. According to Tukey’s HSD multiple comparison test results, in the first postoperative period, the average eGFR score of Group N compared to Group D and Group P was significantly different (*P*<0.001; Table 4). In the second postoperative period, the eGFR average scores of Group N and Group D showed no difference (*P*=0.37), but Group P exhibited a difference (*P*<0.001; Table 5). Evolution of eGFR in the three groups from the first day to the second postoperative day is shown in Figure 1.

**Table 3:** Evaluation of eGFR with ANOVA.

eGFR	Group N	Group D	Group P	P*
Preoperative	52.2±1.54	53.9±1.28	53.7±1.11	0.43
Postoperative day 1	55.4±1.38	53.4±0.9	52.6±1.23	<0.001
Postoperative day 2	53.1±1.04	52.8±0.9	51.3±1.27	<0.001

P*: Postoperative day 1 *vs*. Postoperative day 2.

**Table 4:** Postoperative day 1. *Post-hoc* evaluation of eGFR with Tukey HSD.

Comparison by instructor	Estimated Mean Difference	Standard Error	Tukey Adjusted %95CI	*P*
**NAC**				
Dopamine	2	0.26	1.4;2.6	<0.001
Control	2.8	0.26	2.17;3.4	<0.001
**Dopamine**				
NAC	-2	0.26	-2.6;-1.4	<0.001
Control	0.8	0.26	0.17;1.4	0.009
**Control**				
NAC	-2.8	0.26	-3.4;-2.17	<0.001
Dopamine	-0.8	0.26	-1.4;-0.17	0.009

**Table 5:** Postoperative day 2. *Post-hoc* evaluation of eGFR with Tukey HSD.

Comparison by instructor	Estimated Mean Difference	Standard Error	Tukey Adjusted %95CI	*P*
**NAC**				
Dopamine	0.32	0.24	-2.5;0.9	0.38
Control	1.75	0.24	1.17;2.33	<0.001
**Dopamine**				
NAC	-0.32	0.24	-0.9;0.25	0.38
Control	1.42	0.24	0.85;2	0.009
**Control**				
NAC	-1.75	0.24	-2.33;-1.17	<0.001
Dopamine	-1.42	0.24	-2;-0.85	0.009

**Fig. 2:**
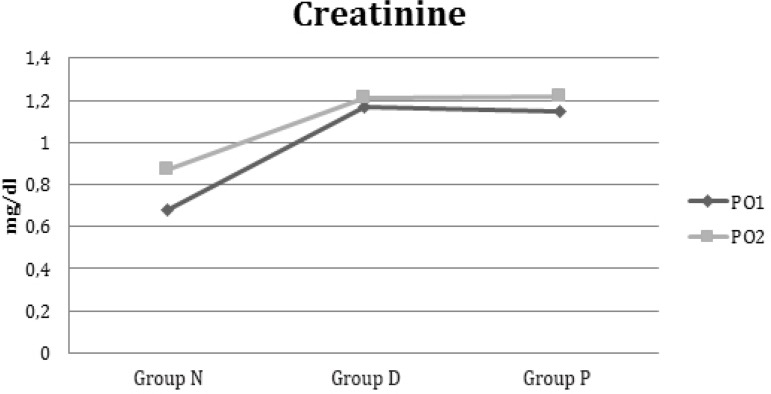
Evolution of creatinine from first postoperative day to second postoperative day in NAC, Dopamine and Placebo Groups.

Preoperative creatinine exhibited no statistically significant differences among Groups N, D, and P (*P*=0.57). In the first and second postoperative days, the drugs used showed statistically significant differences in relation to the creatinine levels (*P*<0.001; Table 6). A *post hoc* test was performed for comparison to determine the source of this difference between the groups. According to Tukey’s HSD multiple comparison test results for the first and second postoperative days, the average creatinine score of Group N and the average creatinine scores of Group D and Group P were significantly different (*P*<0.001; Tables 7 and 8). Evolution of creatinine in the three groups from first day to second postoperative day is shown in Figure 2.

**Table 6:** Evaluation of creatinine with ANOVA.

Creatinine (mg/dl)	Group N	Group D	Group P	*P
Preoperative	1.11±0.05	1.10±0.06	1.09±0.06	0.57
Postoperative day 1	0.68±0.2	1.17±0.18	1.15±0.14	<0.001
Postoperative day 2	0.87±0.15	1.21±0.13	1.22±0.19	<0.001

P*: Postoperative day 1 *vs*. Postoperative day 2.

**Table 7:** Postoperative day 1. Post-hoc evaluation of creatinine with Tukey HSD.

Comparison by instructor	Estimated Mean Difference	Standard Error	Tukey Adjusted %95CI	*P*
**NAC**				
Dopamine	-0.49	0.11	-0.52;-0.46	<0.001
Control	-0.47	0.11	-0.50;-0.44	<0.001
**Dopamine**				
NAC	0.49	0.11	0.46;0.52	<0.001
Control	0.02	0.11	-0.006;0.004	0.18
**Control**				
NAC	0.47	0.11	0.44;0.5	<0.001
Dopamine	-0.02	0.11	-0.05;0.007	0.18

**Table 8:** Postoperative day 2. Post-hoc evaluation of creatinine with Tukey HSD.

Comparison by instructor	Estimated Mean Difference	Standard Error	Tukey Adjusted %95CI	*P*
**NAC**				
Dopamine	-0.34	0.006	-0.35;-0.32	<0.001
Control	-0.33	0.006	-0.34;-0.31	<0.001
**Dopamine**				
NAC	0.34	0.006	0.32;0.35	<0.001
Control	0.007	0.006	-0.09;0.02	0.54
**Control**				
NAC	0.33	0.006	0.31;0.35	<0.001
Dopamine	-0.007	0.006	-0.02;0.009	0.54

## DISCUSSION

Our randomized double-blind study demonstrated that in patients with preexisting mild renal failure undergoing CABG surgery, intravenous NAC had a renoprotective effect, whereas continued low-dose dopamine (renal dose) had no protective effect. NAC caused a statistically significant increase in eGFR and a decrease in creatinine.

Ischemia-reperfusion injury, oxidative stress, and systemic inflammation are factors considered to play a significant role in the development of AKI after cardiac surgery^[5]^. NAC has the potential to be useful in this setting because it reduces the production of oxygen-free radicals^[6]^ and proinflammatory cytokines^[7]^; moreover, it has been shown to reduce the degree of ischemic AKI in animal studies^[8]^. At the same time, it has been illustrated that NAC can reduce the risk of contrast nephropathy in humans^[9]^, while the renoprotective effect of NAC on CPB remains controversial.

Fischer et al.^[10]^ showed that NAC reduced serum creatinine compared with the control group in their study. Moreover, Wijeysundera et al.^[11]^ found that there was preservation of postoperative eGFR in patients with preexisting moderate renal dysfunction undergoing cardiac surgery and a significant reduction in mortality with NAC. In our study, we observed increased creatinine clearance and augmented eGFR.

Although intravenous administration of NAC does not clearly prevent AKI in patients with renal insufficiency undergoing cardiac surgery, Sisillo et al.^[12]^ demonstrated a reduction in the incidence of the serum creatinine concentration. However, this reduction did not reach statistical significance. Furthermore, Ayhan et al.^[13]^ found that two different regimens of NAC in CABG had some beneficial effects in the early the period of the surgery, but failed to demonstrate preventive effects in patients in the late phase. In our study, we demonstrated preventive effects between 24 and 48 hours after surgery.

Santana-Santos et al.^[14]^ showed that the incidence of kidney injury was reduced with NAC in CABG. They investigated two independent markers of kidney injury, namely cystatin C and neutrophil gelatinase associated lipocalin (NGAL). In our study, the criteria used to characterize AKI were serum creatinine and the Cockcroft-Gault equation.

Adabag et al.^[15]^ found that NAC was not significantly associated with serum creatinine levels, the incidence of AKI, hemodialysis requirements, operative mortality, intensive care, or length of hospital stay in patients undergoing cardiac surgery. These patients received oral NAC 600 mg twice daily preoperatively, but the researchers administered NAC orally, and such administration may reduce its effectiveness. Tossios et al.^[16]^. suggested that NAC application should begin before anesthesia induction to yield maximal benefit of its reactive oxygen species (ROS)-scavenging properties. Considering that the NAC is estimated to have a short half-life, at 2.2 hours^[17]^, in this study, 50 mg/kg of NAC was administered as the loading dose in 100 cc of 0.9% NaCl for 15 minutes; following this 20 mg/kg/h of NAC was given as an infusion in 100 cc of 0.9% NaCl during the operation.

Hyninen et al.^[18]^ demonstrated that NAC does not decrease the amount of kidney injury occurring in patients with normal preoperative renal function undergoing abdominal aortic surgery. In addition, Sucu et al.^[6]^ found that intravenous NAC decreased the pump-induced oxido-inflammatory response during CPB. In aortic surgery, it may result in kidney injury, and this may have different mechanisms, with the exception oxido-inflammatory damage.

Haase et al.^[19]^ concluded that a high dose (300 mg/kg intravenously) of NAC was no more effective than a placebo in attenuating CPB-related acute renal failure in high-risk cardiac surgery patients. Theoretically, high-dose NAC may have been excessive, and paradoxically, it may have diminished the level of radical oxygen species, thereby attenuating their potentially positive role in the regulation of intracellular signaling^[20]^.

There has been no consensus on the identification of the most effective route and dose of NAC administration associated with significant renal protection in patients undergoing CABG. However, NAC is an inexpensive, relatively safe, well-tolerated, and widely used drug.

Although whether the use of low-dose dopamine protects against acute renal failure is controversial, renal-dose dopamine is still one of the most frequently used prophylactic measures to prevent renal dysfunction in patients. Its potentially advantageous effects include an increase in renal blood flow via activation of dopaminergic receptors in the renal vasculature^[4]^.

In several controlled trials with dopamine, improvements in several renal function parameters have been reported^[21]^. However, Lassnigg et al.^[22]^ showed that infusion of dopamine was ineffective for renal protection. Similarly, in our study, we demonstrated that dopamine was not effective for renal protection. At the same time, low-dose dopamine was shown to reverse periods of oliguria after CPB in an uncontrolled study^[23]^. In our study, urine output increased in Group D compared with Group N and Group P.

Limitations

This study had several limitations. The diagnosis of acute renal failure was primarily based on an increase in serum creatinine, which may not accurately reflect true changes in GFR^[24]^. Moreover, eGFR is a surrogate outcome that does not accurately represent the true GFR in the non-steady state of AKI^[25]^; however, alternative options are limited. Newer urinary biomarkers, such as cystatin C or NGAL, may be more sensitive for identifying kidney damage^[26]^. At present, however, serum creatinine is the cheapest and most broadly accepted marker of kidney function^[27]^.

If NAC reduces creatinine generation by protecting the creatinine kinase activity, our study would not have been adequate to explore this effect. However, it has recently been suggested that NAC may decrease serum creatinine concentration without affecting renal function^[28]^.

## CONCLUSION

We found that intravenous prophylactic use of NAC had a protective effect on renal function. In contrast, the application of renal-dose dopamine did not have a protective effect in patients with preexisting moderate renal failure.

**Table:** 

Authors’ roles & responsibilities	
OFS	Conception and study design; analysis and/or data interpretation; statistical analysis; manuscript redaction or critical review of its content; final manuscript approval
FG	Conception and study design; analysis and/or data interpretation; statistical analysis; manuscript redaction or critical review of its content; final manuscript approval
YY	Conception and study design; analysis and/or data interpretation; statistical analysis; manuscript redaction or critical review of its content; final manuscript approval
DC	Conception and study design; analysis and/or data interpretation; statistical analysis; manuscript redaction or critical review of its content; final manuscript approval
EG	Conception and study design; analysis and/or data interpretation; statistical analysis; Manuscript redaction or critical review of its content; final manuscript approval
HO	Conception and study design; analysis and/or data interpretation; statistical analysis; manuscript redaction or critical review of its content; final manuscript approval
TO	Conception and study design; analysis and/or data interpretation; statistical analysis; manuscript redaction or critical review of its content; final manuscript approval
TK	Conception and study design; analysis and/or data interpretation; statistical analysis; manuscript redaction or critical review of its content; final manuscript approval
